# Supplementary material for: The genome sequence of the Gold Spangle, Autographa bractea (Denis & Schiffermüller), 1775 (Lepidoptera: Noctuidae)
Source: Wellcome Open Res. 2026 Jan 23;11:57. [Version 1] doi: 10.12688/wellcomeopenres.25784.1 (PMC12910202; doi:10.12688/wellcomeopenres.25784.1)
Supplement: Peer review history

# Reviewer report: Reviewer response for version 1

**DOI:** 10.21956/wellcomeopenres.28385.r146432

**Published:** 2026-02-16

**Authors:** Xue Qing

**Affiliations:** 1 Nanjing Agricultural University, Nanjing, China

This data presents the first complete genome sequence of the Gold Spangle moth, Autographa bractea, as part of the Project Psyche initiative. The genome was assembled from a single male specimen using PacBio HiFi sequencing and Hi-C scaffolding. 

 This genome note is exemplary in both technical rigor and clarity. It provides a valuable genomic resource for Lepidoptera research and contributes to the broader goals of the Tree of Life. The detailed protocols, use of state-of-the-art tools, and open data practices make it a model for future genome reports. Overall, this manuscript can be indexed with minor revision. below are my comments:

 1. the abstract could briefly mention the evolutionary or conservation significance of this genome to broaden its impact.

 2. Figure legends could be more self-explanatory, allowing readers to understand them independently. Some specialized terms (e.g., "Merian elements") could include a short explanation for non-specialists.

 3. adding a brief discussion on how this genome compares to related species would enhance its value for comparative genomics.

Are sufficient details of methods and materials provided to allow replication by others?

Yes

Is the rationale for creating the dataset(s) clearly described?

Yes

Are the datasets clearly presented in a useable and accessible format?

Yes

Are the protocols appropriate and is the work technically sound?

Yes

Reviewer Expertise:

comparative genomics of invertbrates

I confirm that I have read this submission and believe that I have an appropriate level of expertise to confirm that it is of an acceptable scientific standard.

# Reviewer report: Reviewer response for version 1

**DOI:** 10.21956/wellcomeopenres.28385.r146428

**Published:** 2026-02-05

**Authors:** Gen Morinaga

**Affiliations:** 1 University of Calgary, Calgary, Alberta, Canada

In the genome note titled “The genome sequence of the Gold Spangle, *Autographa bractea *(Denis & Schiffermuller), 1775 (Lepidoptera: Noctuidae)”, Cornet et al., sequence the genome of an individual Gold Spangle moth ( *A. bractea*) using the PacBio HiFi sequencing platform. Furthermore, they combine these sequences with Hi-C sequencing of the same individual to produce haplotype representation assemblies, both of which exhibit high gene content completeness, with the primary haplotype also exhibiting a high degree of contiguity after scaffolding and manual curation. Lastly, they identify the sex chromosome and assemble and annotate the mitochondrial genome. They “painted” (i.e., positionally identified) Merian elements on each chromosome and presented it in a very nice figure.

 I believe the work presented is done to a high standard using state-of-the-art bioinformatic tools. I especially appreciated the detailed methods. The writing is generally very clear, and though I prefer using an “active voice” (e.g., “The genome was assembled…” vs. “We assembled the genome…”), the use of the “passive voice” in this work is not a hinderance to the reader.

Are sufficient details of methods and materials provided to allow replication by others?

Yes

Is the rationale for creating the dataset(s) clearly described?

Yes

Are the datasets clearly presented in a useable and accessible format?

Yes

Are the protocols appropriate and is the work technically sound?

Yes

Reviewer Expertise:

Evolutionary biology; Genomics; Comparative Genomics; Bioinformatics

I confirm that I have read this submission and believe that I have an appropriate level of expertise to confirm that it is of an acceptable scientific standard.
